# Editorial

**Published:** 2011-04-15

**Authors:** Usha Mohan Das

**Affiliations:** Editor-in-Chief

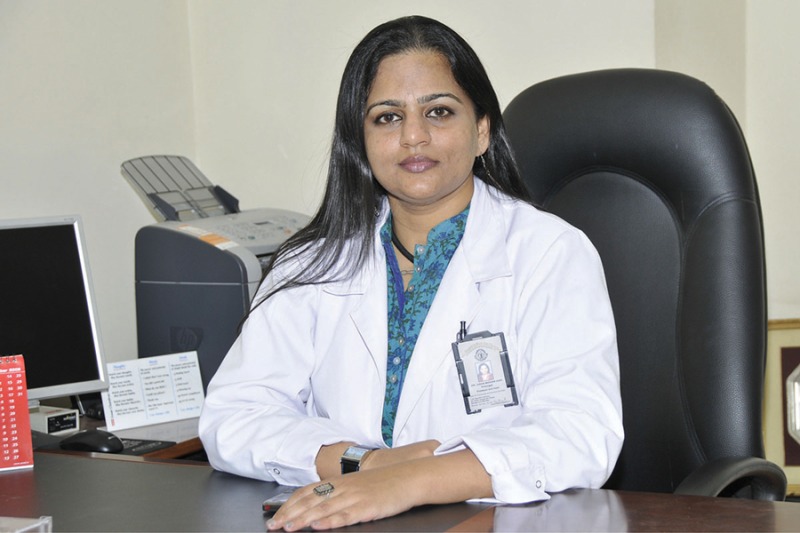



**Living the Vision ...**

When John F Kennedy, the late President of the United States, was touring the NASA (National Aeronautics and Space Administration) and its operations, he walked by a man with cleaning supplies and asked him, “What he did at NASA?” The Janitor responded that he was helping to put a man on the moon.

Do we live our vision in pediatric dentistry in a similar manner? In my opinion, holding vision and work together makes it a reality. Do we individually and collectively provide and create an environment where the science of pediatric dentistry and its specialists are able to achieve their full potential and realize their dreams? How could we take this wonderful science and practice forward and increase its accessibility to each and every needy patient?

Do we stop to think what a member’s full potential could be? We have many societies/associations in the world, which foster and nourish specialized pediatric dental surgeons. How many of us know or truly translate into action our dreams for better oral health care for the children and young adults in the society? If we embody the vision of pediatric dentistry, we will ensure that our speciality thrives and grows.

As a scientific community, it is very important to nourish each and every member’s dream and not work as individuals. It is only action with vision that would make a difference globally to the oral health care needs of children being fulfilled through pediatric dentistry.

Just as the NASA Janitor worked to fulfill the vision statement of his organization, we can surely make a difference in the lives of the little children we serve with a vision. Only when this happens globally on a single united platform, we are truly pediatric dental surgeons achieveing greatness together.

**Usha Mohan Das**

Editor-in-Chief

e-mail: ushymohandas@gmail.com

